# Home phototherapy for neonatal hyperbilirubinemia: a mixed methods systematic review and meta-analysis

**DOI:** 10.1038/s41390-025-04313-5

**Published:** 2025-07-26

**Authors:** Jessie Spaan, Lauren E. H. Westenberg, Erwin Ista, Berthe A. M. van der Geest, Irwin K. M. Reiss, Sten P. Willemsen, Christian V. Hulzebos, Jasper V. Been

**Affiliations:** 1 Department of Neonatal and Pediatric Intensive Care, Division of Neonatology, Erasmus MC Sophia Children’s Hospital, University Medical Center Rotterdam, Rotterdam, the Netherlands; 2 Department of Neonatal and Pediatric Intensive Care, Division of Pediatric Intensive Care, Erasmus MC Sophia Children’s Hospital, University Medical Center Rotterdam, Rotterdam, the Netherlands; 3 Department of Internal Medicine, Division of Nursing Science, Erasmus MC, University Medical Center Rotterdam, Rotterdam, the Netherlands; 4 Department of Biostatistics, Erasmus MC, University Medical Center Rotterdam, Rotterdam, The Netherlands; 5 Department of Obstetrics and Gynecology, Erasmus MC Sophia Children’s Hospital, University Medical Center Rotterdam, Rotterdam, the Netherlands; 6 Department of Pediatrics, Division of Neonatology, Beatrix Children’s Hospital, University Medical Center Groningen, Groningen, the Netherlands

## Abstract

**Introduction:**

To promote home phototherapy adoption, clearer insights are needed on current evidence and the population most likely to benefit.

**Methods:**

Six online databases were searched until 20 January 2025. Studies were included that reported on (cost-)effectiveness, safety, and/or implementation barriers and facilitators of home phototherapy. Two reviewers independently extracted data and assessed bias using the Mixed Methods Appraisal Tool. Data were pooled via random-effects meta-analysis. Barriers and facilitators were analyzed using the Theoretical Domains Framework. GRADE was used to assess evidence quality.

**Results:**

Thirty-one studies were included, representing 5059 neonates, 869 healthcare professionals, and 478 parents. Risk of bias was moderate. Hospital (re)admission following home phototherapy was 3.5% (95% CI, 2.2–5.3%). No difference was found in treatment duration (mean difference = +4.9 h, 95% CI –8.0 to +17.9) and daily decline in bilirubin (mean difference = +5.7 μmol/L, 95% CI −2.6 to +13.9). No studies evaluated cost-effectiveness. Key facilitators included eligibility screening and parental education, while key barriers included bilirubin measurement, healthcare professionals’ concerns about homecare consequences, and parental insecurity. Quality of evidence was rated low for all outcomes.

**Conclusion:**

Evidence suggests home phototherapy can be an alternative to in-hospital treatment for hyperbilirubinemia in near-term neonates. Clinical recommendations are limited due to low-certainty evidence.

**Impact:**

- Home phototherapy can be a safe and effective alternative to in-hospital treatment for low-risk neonates with hyperbilirubinemia. Strength of this recommendation is low.
- Our study is the first to systematically synthesize parent/caregiver and healthcare professional perspectives on home phototherapy using the Theoretical Domains Framework.
- Key facilitators included eligibility screening and parental education, while key barriers included concerns about home care consequences by healthcare professionals and parental insecurity. Addressing these barriers through improved support and accurate monitoring is recommended.

## Introduction

Neonatal jaundice is caused by elevated levels of unconjugated bilirubin, affecting 60% of full term neonates and up to 80% of preterm neonates.^1,2^ Depending on screening practices and treatment thresholds, which vary in different guidelines, 4–9% of neonates born after 35 weeks of gestation will develop clinically significant hyperbilirubinemia.^2–4^ Phototherapy continues to be the primary treatment to decrease unconjugated serum bilirubin levels and is applied in-hospital in most settings.^5^ The light used during phototherapy treatment converts bilirubin into water-soluble compounds. It allows the neonate’s body to eliminate it through urine and stool. This process helps prevent bilirubin from reaching dangerously high levels, which could otherwise result in brain damage (kernicterus) if untreated.^6,7^

Currently, severe hyperbilirubinemia is a primary cause of hospitalization within the first week of the neonate’s life, accounting for up to 35% of hospital (re)admissions in high-resource settings.^2^ Home phototherapy has the potential to avoid hospital admissions, reduce associated costs, and promote parent-child bonding. Despite this, home phototherapy for neonatal hyperbilirubinemia is not commonplace. Barriers to implementation, such as the lack of a uniform protocol and concerns about potential consequences of home-based care, may arise at various levels.^8,9^ In the last decade, five systematic reviews including two meta-analyses have evaluated home phototherapy, each focusing on various outcomes or study types, with contradicting results.^10–14^ Moreover, international guidelines use different criteria for selecting neonates and parent(s)/caregiver(s) for home phototherapy.^15^ In order to facilitate widespread adoption of home phototherapy in a safe and effective manner, clearer insights are needed on the resulting recommendations arising from the current existing evidence and the population that home phototherapy may be best utilized. Thus, a clear overview is needed for presenting evidence summaries, using a systematic approach offered by the GRADE guideline.^16,17^

We therefore conducted an updated systematic review, including meta-analyses, to provide the most comprehensive synthesis to date on home phototherapy use for neonatal hyperbilirubinemia. This includes summarizing quantitative and qualitative evidence on (cost-)effectiveness, safety, inclusion criteria, and parental and health care professionals’ experiences. We assessed the certainty of the results for each outcome. This work will facilitate further consideration of home phototherapy in international guidelines on the management of hyperbilirubinemia in neonates.

## Methods

This review is reported according to the Preferred Reporting Items for Systematic Reviews and Meta-Analyses (PRISMA) guidelines (Supplementary Table S1).^18,19^ Our review was prospectively registered in PROSPERO, registration number: CRD42023386798.^20^

### Study inclusion

We included primary quantitative, qualitative, and mixed-methods studies that assessed the effectiveness and/or safety of home phototherapy for neonatal hyperbilirubinemia, or that provided insight into parental or health care professionals’ experiences, or into barriers and/or facilitators to its implementation. The outcomes of interest are summarized below. Case reports, commentaries, reviews, study protocols, and conference abstracts were excluded. In-hospital phototherapy was considered the reference treatment. Studies evaluating neonates born after 35 weeks gestation, aged 0 to 28 days, were considered eligible.

### Outcomes of interest

We divided the outcomes of interest into three groups: (1) effectiveness, (2) safety, (3) barriers and facilitators to implementation. First, effectiveness outcomes were defined as following: (1.1) hospital (re)admission rate, (1.2) treatment duration, (1.3) daily bilirubin reduction, (1.4) and health care costs. Second, safety outcomes included: (2.1) any adverse events of phototherapy treatment, (2.2) the need for exchange transfusion, (2.3) reported acute bilirubin encephalopathy (ABE), (2.4) and reported Kernicterus Spectrum Disorder (KSD). Finally, (3.1) the barriers and (3.2) facilitators to implementation according to parents and healthcare professionals.

### Search strategy and selection criteria

The following six online databases were systematically searched for relevant studies: MEDLINE/PubMed, Embase, Cumulative Index to Nursing and Allied Health Literature (CINAHL), Cochrane Library, Web of Science and Google Scholar. We used a combination of Medical Subject Headings (MeSH) terms and free text to search for potentially eligible studies. A tailor-made search strategy for each database was designed in close collaboration with an experienced biomedical information specialist (Supplementary Table S2). There were no restrictions imposed on language, geographical location, publication date, or timeframe of the study. The search was updated on 20 January 2025 for all databases to include studies published since the initial search (8 June 2022). In addition, the reference lists of included studies and their citations (through Google Scholar) were screened to identify additional relevant studies that may have been missed. All records identified were downloaded into EndNote™ and duplicates were deleted. Two reviewers (JS and LEHW) independently screened titles and abstracts to select potentially relevant records. When a record was considered relevant or when the title/abstract was deemed insufficiently informative for deciding inclusion/exclusion, the full text was retrieved and evaluated. Discrepancies and uncertainties at any stage in this selection process were resolved through discussion with a third reviewer (JVB) until consensus was reached.

### Data extraction

Data extraction was performed independently by two reviewers (JS and LEHW) using a standardized form specifically designed for this review (Supplementary Table S3). Subsequently, the reviewers shared their results and created a final extraction table. Any disagreement was discussed and resolved through consensus, if necessary with a third reviewer (JVB). The information extracted included characteristics of the study, methods, and study population, as well as the relevant outcomes (effectiveness, safety, and on barriers and facilitators to implementation according to healthcare professionals and parents). For the effectiveness and safety, numerical data were extracted. For the barriers and facilitators to implementation according to parents and healthcare professionals, themes, authors’ interpretations, and participants’ quotations of the qualitative studies, as well as data from quantitative studies were extracted. In case the data in the original publications were not sufficiently detailed or unclear, we contacted the authors for clarification and additional information.

### Risk-of-bias assessment of included studies

Two reviewers independently (JS and LEHW) assessed risk of bias for each study using the Mixed Methods Appraisal Tool (MMAT).^21^ MMAT is designed for mixed-methods systematic reviews and appraises methodological aspects of five study designs: qualitative research, randomized studies, non-randomized studies, descriptive studies, and mixed methods studies. Any disagreement was resolved through discussion, and when necessary, by involving a third reviewer (JVB). Risk-of-bias assessment was not used to exclude any studies in this review and instead used to assess confidence in the evidence.

### Grading the quality of evidence of outcomes of interest

Two reviewers independently (JS and LEHW) used the Grading of Recommendations Assessment, Development and Evaluation (GRADE) to assess the certainty of the synthesized results for the outcomes of interest.^16,17^ We pre-selected the following outcomes for the GRADE assessment: (1) effectiveness: treatment duration, (2) effectiveness: daily bilirubin reduction, (3) effectiveness: hospital (re)admission rate, (4) effectiveness: healthcare costs, and (5) safety: any adverse events of phototherapy treatment.

### Data synthesis

#### Meta-analyses

Meta-analysis was possible for the following outcomes: hospital readmission rates, daily decline of bilirubin levels, and treatment duration. Heterogeneity of the findings was assessed using the I^2^ statistic. An I^2^ > 75% was considered as substantial heterogeneity. Random effects models were used to calculate pooled estimates and the corresponding 95% confidence intervals (CI). For hospital readmission rates, the standard continuity correction (adding ½ to the number of participants with and without an event) was applied in case a study group had zero events. In case included articles reported on the same study population, this study population was then considered only once to prevent duplication in the analyses. Furthermore, subgroup analyses were conducted to evaluate whether factors as bilirubin levels, term status, and age at phototherapy initiation impacted the readmission rate. For the daily decline of bilirubin levels and the treatment duration, only studies with a control group (in-hospital phototherapy) were included in the meta-analyses. Analyses were performed using R (version 4.3.1.) packages meta version 4.9-7 and metaphor 2.0-0. If necessary, we converted bilirubin levels from mg/dL to μmol/L by applying a conversion factor of 17.1.

#### Barriers and facilitators according to healthcare professionals and parents

To further explore barriers and facilitators to implementation of home phototherapy, both qualitative and quantitative findings were synthesized based on the Theoretical Domains Framework (TDF).^22^ TDF provides a theory-based approach to identify determinants. This framework consists of 14 domains that integrate constructs from multiple theories in relation to behavior change: 1) knowledge; 2) skills; 3) social/professional role and identity; 4) beliefs about capabilities; 5) optimism; 6) beliefs about consequences; 7) reinforcement; 8) intentions; 9) goals; 10) memory, attention, and decision processes; 11) environmental context and resources; 12) social influences; 13) emotion; and 14) behavioral regulation.^22^ Two reviewers (JS and LEHW) discussed the studies to reach consensus regarding the identification and coding of themes. Discrepancies were resolved by discussion with an implementation expert (EI) until consensus was reached.

## Results

**Fig. 1:**
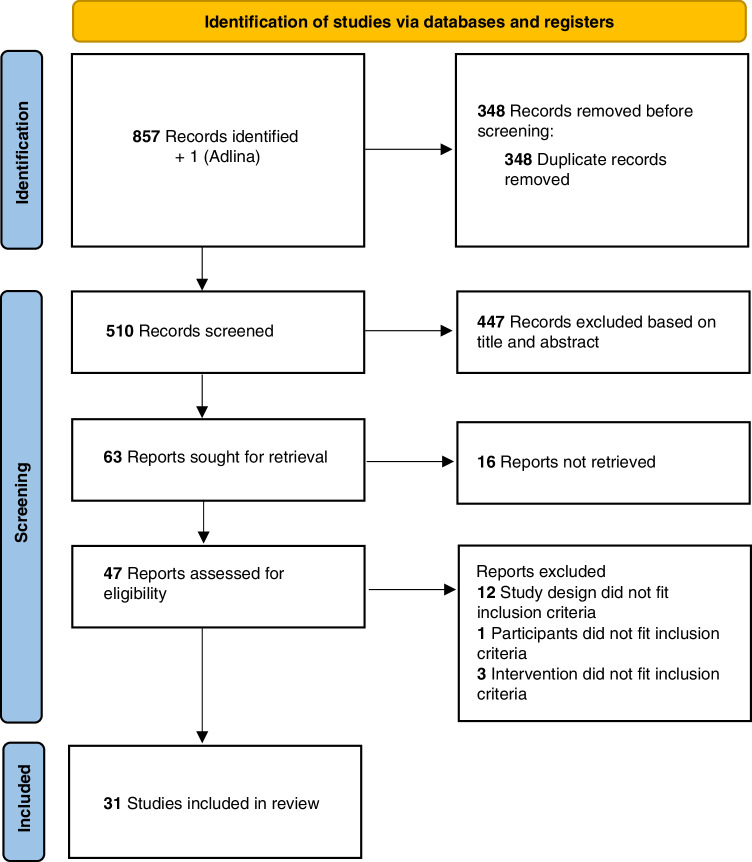
The Preferred Reporting Items for Systematic Review and Meta-Analyses flow chart of screening and selection of studies, with reasons for exclusion based on full text review.^18^ PRISMA flow diagram of the literature search and selection process.^18^

A flow diagram of the study selection process is presented in Fig. 1. We identified 857 records from database searches, and after removing duplicates, 510 studies were screened based on their titles and abstracts. Following this initial screening, 63 potentially relevant studies were retrieved for full text assessment. Additionally, one more relevant study was identified through reference searching. A total of 31 studies were included in this systematic review.

### Study characteristics

Table 1 lists the main study characteristics of the 31 included studies, representing 5059 neonates, 869 health care professionals, and 478 parents. This compilation comprises five randomized controlled trials,^23–27^ 10 non-randomized studies,^28–37^ 13 quantitative descriptive studies,^8,9,38–48^ one mixed-methods study,^49^ and two qualitative studies.^50,51^ Five studies have examined data from two study populations, with one study population being explored in three papers,^24–26^ and the other in two papers respectively.^23,27^ Eleven studies were performed in North America,^29,33–35,39–45^ nine in Europe,^9,24–26,28,32,47,48,51^ eight in Asia,^23,27,30,36–38,46,49^ and three in Oceania.^8,31,50^ Studies were published between 1984 and 2024.

**Table 1:** Summary of characteristics of included studies.

Author and year	Country	Study design	Study population	Inclusion criteria	Exclusion criteria	Effectiveness	Safety	Parental experiences	HCP’s experiences	Costs	MMAT
Adlina et al. 2007^38^	Malaysia	Quantitative descriptive study	Neonates *N *= 1297	Not reported	Not reported	✔					40%
Anderson et al. 2023^50^	Australia	Qualitative study	Healthcare providers *N *= 9	Healthcare professionals currently working in the NICU Department.	Junior doctors, nurses, and other healthcare professionals working in NICU.				✔		60%
Chang et al. 2020^39^	USA	Quantitative descriptive study	Neonates *N *= 1324	GA ≥ 35 weeks.	Age at start PT > 14 days; Conjugated bilirubin level > 34.2 μmol/L.	✔					100%
Cnossen et al. 2024^9^	Netherlands	Quantitative desriptive study	Healthcare providers Pediatricians and community midwives *N *= 106 Maternity care assistants *N *= 515	Healthcare professionals working in the perinatal care with and without home PT experience.					✔		100%
Coquery et al. 2022^28^	France	Non-randomized study	Neonates *N *= 100	GA ≥ 35 weeks; Unconjugated hyperbilirubinemia.	Conjugated bilirubin level elevated; Unknown etiology of jaundice; Indication for exchange blood transfusion or 50 μmol/L below the exchange transfusion threshold; Weight loss > 10%; Other associated pathologies (infection or malformation).	✔					80%
Dortch et al. 1986^40^	USA	Quantitative descriptive study	Neonates *N *= 41	Age at start PT > 24 hours; Weight > 2000 grams; Bilirubin level > 239.4 μmol/L.	Sepsis; Progressing anemia.	✔		✔	✔	✔	40%
			Mothers *N *= 11								
Eggert et al. 1985^29^	USA	Non- randomized study	Neonates HPT *N *= 62 IPT *N *= 55	Age at start PT > 24 hours Birth weight > 2270 g Infant is clinically well Total bilirubin level allowed adequate margin of error for a trial of HPT; Parents capable of managing HPT.	Not reported	✔	✔			✔	100%
Ellis et al. 1985^41^	USA	Quantitative descriptive study	Not reported	Referral was made by the pediatrician; Nurses who visit the home assessed the suitability of parents.	Not reported	✔		✔	✔	✔	0%
Grabert et al. 1985^42^	USA	Quantitative descriptive study	Neonates *N *= 260 Parents *N *= 20	Not reported	Not reported	✔	✔	✔	✔	✔	100%
			Healthcare providers *N *= 200								
Hamelin et al. 1998^43^	Canada	Quantitative descriptive study	Neonates *N *= 26	GA > 37 weeks; Healthy; Age at start PT > 24 h; Feeding well by breast or bottle (with no evidence of dehydration); Adequate home and parental environment.	No physical abnormalities; No significant weight loss; Stooling and voiding by 24 hours of age.	✔		✔		✔	60%
Jackson et al. 2000^8^	Australia	Quantitative descriptive study	Neonates *N *= 32 Parents *N *= 28	Healthy; Feeding well; Serum bilirubin was in the PT range; Parents opted to receive PT at home, rather than in hospital.	Not reported	✔		✔		✔	100%
Jahan et al. 2023^49^	Bangladesh	Mixed Method study	Healthcare providers *N *= 10 Mothers with neonate receiving HPT *N *= 11 Mothers with neonate who did not receive HPT *N *= 3 Parents *N *= 16 Grandparents *N *= 14	Not reported	Not reported			✔	✔		20%
Kavosi et al. 2023^30^	Iran	Non-randomized study	Neonates HPT *N *= 90 IPT *N *= 90	Term infants; Total bilirubin between 171 and 307.8 μmol/L; Normal weight (2500–4000 grams); Age at start PT > 24 h.	Premature infants (weight under 2500 grams and fetal age under 37 weeks); Infants with congenital anomalies; Infants with blood disorders and infections; Direct bilirubin above 25.65 μmol/L.					✔	80%
Khajehei et al. 2022^31^	Australia	Non- randomized study	Neonates HPT *N *= 400 IPT *N *= 241 Parents *N *= 130	GA ≥ 35 weeks; Hospital discharge between 4 and 96 hours after birth; Family compliant (language and social).	Not reported	✔		✔	✔	✔	80%
Meropol et al. 1993^44^	USA	Quantitative descriptive study	Community pediatricians *N *= 29	Not reported	Not reported				✔		100%
Namnabati et al. 2019^23^	Iran	Randomized controlled trial	Mothers HPT *N *= 32 IPT *N *= 32	Term infants; Age at start PT > 3 days; Weight > 2500 gram; Physician’s confirmation of PT need; Indirect bilirubin >239.4 μmol/L and <307.8 μmol/L); Negative Coombs test; No increase in direct bilirubin; Absence of risk factors like lethargy, rejection of breastfeeding, fever, blood type incompatibility, polycythemia, favism, anemia and history of severe neonatal jaundice in the family.	Parents unwilling to continue the study; Occurrence of a stressful event during the study for parents.			✔			60%
Noureldein et al. 2021^32^	UK	Non-randomized study	Neonates HPT *N *= 100 IPT *N *= 50 Parents *N *= 100	GA ≥ 35 weeks; Age ≥ 48 h; Weight ≥ 2000 gram; Bilirubin level < 50 umol/L above treatment threshold; Parents able to converse in English, follow instructions regarding to the use of PT equipment and having a satisfactory home environment	Severe haemolytic jaundice.	✔		✔			100%
Orringer et al. 2022^33^	USA	Non- randomized study	Neonates *N* = 159	Neonates with TSB levels within 0.1 to 0.3 mg/dl below inpatient treatment threshold.	Neonates not born at affiliated center; Received PT prior to first clinic visit; No TSB level drawn in the clinic.	✔					80%
Pettersson et al. 2021^24^	Sweden	Randomized controlled trial	Neonates HPT *N *= 78 IPT *N *= 69	GA > 36 weeks; TSB > 300 μmol/L between 48 and 72 h or TSB > 350 μmol/L after 72 h of age; Parents capable to perform the therapy and accepting to turn to hospital for daily check-up.	Blood group incompatibility (defined as a positive direct antiglobulin test); TSB > 425 μmol/L Asphyxia; Weight loss > 10%; Ongoing infection or any other severe illness; Parents did not speak Swedish or the PA felt they would not be able to handle home PT.	✔	✔				60%
Pettersson et al. 2021 (2)^25^								✔			60%
Pettersson et al. 2023^26^										✔	60%
Pettersson et al. 2022^51^	Sweden	Qualitative study	Parents *N *= 15	Not reported	Not reported			✔			100%
Rogerson et al. 1985^45^	USA	Quantitative descriptive study	Neonates *N *= 86	Not reported	Not reported	✔		✔			60%
Saeid et al. 2023^46^	Iran	Quantitative descriptive study	Neonates *N *= 203	Term infants; Age at start PT > 24 h; Normal weight (2500–4000 g).	Premature infants (weight under 2500 grams and fetal age under 37 weeks); Infants with congenital anomalies; Infants who had to receive serum; Infants who had congenital anomalies; History of PT treatment or blood transfusions; Infants with blood disorders, hemolysis and infection.	✔	✔				80%
Sardari et al. 2019^27^	Iran	Randomized controlled trial	Neonates HPT *N *= 32 IPT *N *= 32	Term infants with a total bilirubin level between 239.4 μmol/L and 307.8 μmol/L; Age at start PT > 3 days; Weight > 2500 grams; No risk symptoms such as mother infant blood incompatibility, polycythemia, Coombs positive, anemia and no history of severe neonatal jaundice in the family.	Not reported	✔	✔				40%
Schuman et al. 1990^34^	USA	Non- randomized study	Neonates Fibreoptic *N *= 22 Conventional *N *= 26 Parents *N *= 36	Term infants with no preceding in hospital PT; Negative Coombs test; Home PT ordered by the infant’s pediatrician.	Infants who failed to complete home PT and had treatment completed on an inpatient basis.	✔		✔			100%
Slater et al. 1984^35^	USA	Non- randomized study	Neonates HPT *N *= 25 IPT *N *= 33	GA > 37 weeks; Weight of 2500 to 4000 gram; Five minute Apgar score ≥ 7; Normal findings on physical examination; Actively feeding infant; Stooling and voiding by 24 h of age; Age at start PT > 48 h of age but <7 days Direct bilirubin <1.5 mg/100 mL and no evidence of Rh isoimmunization by direct Coombs and hematocrit; Adequate home and parent environment as evaluated by study staff.	Pathological causes of jaundice; No adequate home support network; Language barriers; Lacked telephone communication.	✔	✔	✔		✔	100%
Thakkar et al. 2019^47^	UK	Quantitative descriptive study	Neonates *N *= 10	Receiving PT in hospital for ≥48 h with stable or falling bilirubin levels; Likely to require prolonged PT; Weight loss <10%; Feeds established; No contra-indications to discharge; Infant’s address within the Trust’s catchment area.	Not reported	✔	✔	✔		✔	60%
Yilmaz et al. 2015^36^	Turkey	Non- randomized study	Neonates HPT *N *= 25 IPT *N *= 25	GA ≥ 37 weeks; Birth weight ≥ 2500 g; Age at start PT > 4 days; Non-hemolytic jaundice; Non-cholestatic jaundice (defined as direct bilirubin <20% of total serum bilirubin levels).	Serum indirect bilirubin level higher than 20 mg/dL	✔					80%
Zainab et al. 2004^37^	Malaysia	Non-randomized study	Neonates HPT *N *= 18 IPT *N *= 18	GA > 37 weeks; Birth weight 2500–4000 g; Apgar score 5 min > 7; Normal findings on physical examination; Actively feeding infant; Stooling and voiding by 24 h; Age at start PT: 2 to 7 days; No G6PD deficiency, No ABO incompatibility negative Coombs test; Adequate home and parental environment as evaluated by study staff.	Not reported	✔	✔	✔		✔	100%
Zanardo et al. 2021^48^	Italy	Quantitative descriptive study	Neonates *N *= 30 Parents *N *= 30	Near-term and term infants; Body weight decrease of less than 10%; No Rh or ABO iso-immunisation as indexed by a positive direct antiglobulin test; No IUGR and serious congenital malformations; No syndromes or other congenital diseases that could affect the outcome measure; Parents capable to perform PT at home.	Not reported	✔		✔			80%

We converted units of bilirubin levels from mg/dl to μmol/L using a conversion factor of 17.1 (i.e. 1 mg/dl = 17.1 umol/L).

*USA* United States of America, *UK* United Kingdom, *GA* gestational age, *IUGR* Intrauterine Growth Restriction, *ABO* ABO blood group system, *Rh* Rhesus factor, *PT* phototherapy, *HPT* home phototherapy, *IPT* in-hospital phototherapy, *TSB* total serum bilirubin, *NICU* Neonatal Intensive Care Unit, *HCP’s* healthcare professionals’, *MMAT* mixed methods appraisal tool.

The home phototherapy programs in the included studies varied in several key characteristics, including different inclusion criteria for home phototherapy program. The most reported characteristics determining eligibility were gestational age and postnatal age at the initiation of phototherapy. Some studies included neonates with a gestational age from 35 weeks,^4,28,31,32,48^ others 36 weeks,^24^ and some 37 weeks.^23,30,34,35,43^ The lower bound of postnatal age at start of phototherapy varied between 24 h,^29,30,40,43,46^ 48 h,^24,32,35,37,46,47^ and 72 h,^23,27^ as summarized in Table 1. Characteristics of the home phototherapy devices used in the included studies can be found in Supplementary Table S4.

### Risk of bias of the studies

Detailed critical appraisals of included studies are available in Supplementary Table S5. In the five randomized controlled trials, multiple studies inadequately reported details regarding randomization and blinding. All quantitative non-randomized studies were rated as having a low risk of bias. Risk of bias of quantitative descriptive studies varied, often affected by factors such as the lack of reported statistical analysis techniques and unclear risks of nonresponse bias. In the mixed-methods study, the rationale for using the methodology, integration, and interpretation of the qualitative and quantitative data was missing. The qualitative studies were rated as moderate to low risk of bias. Overall, risk of bias of most studies was rated as moderate.

### Primary outcome - effectiveness

Data on effectiveness of home phototherapy were available in eight (non)-randomized studies and in 13 single-arm studies (Supplementary Table S6).

#### Hospital readmission rates following home phototherapy

**Fig. 2:**
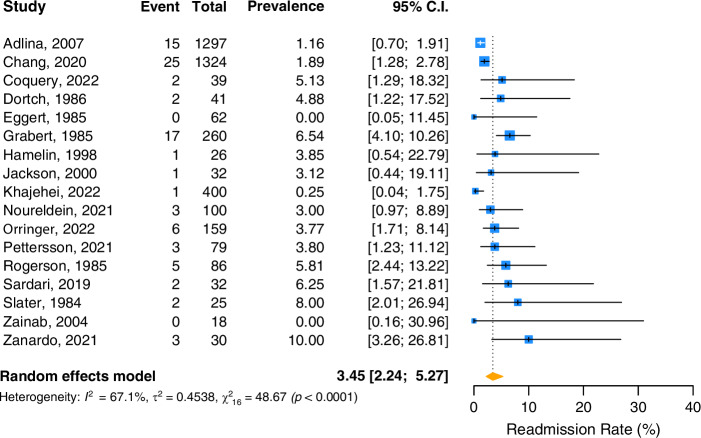
Forest plot meta-analysis of hospital (re)admission rate. CI confidence interval.

Data from 17 studies were included in this meta-analysis.^8,24,27–29,31–33,35,37–40,42,43,45,48^ The mean hospital (re)admission rate across these studies was 3.5% (95% CI) 2.2–5.3; *I*^2^ = 67%; 4010 participants) (Fig. 2). Reasons for readmission included: reduced exposure duration to phototherapy due to language and cultural barriers, non-compliance with the treatment protocol, parental anxiety, and clinical concerns for the neonate, such as temperature instability.

A subgroup analysis was conducted on studies that included only neonates with bilirubin levels above the phototherapy threshold at initiation of treatment, showing a readmission rate of 3.7% (95% CI = 2.3–5.8; 1389 participants). Similarly, a subgroup analysis of studies that included only term neonates showed a readmission rate of 4.8% (95% CI = 2.5–12.7; 101 participants). For studies including only neonates above 48 h of postnatal age at initiation of phototherapy, the readmission rate was 4.4% (95% CI = 2.4–7.9; 254 participants).

#### Treatment duration in home versus in-hospital phototherapy

**Fig. 3:**
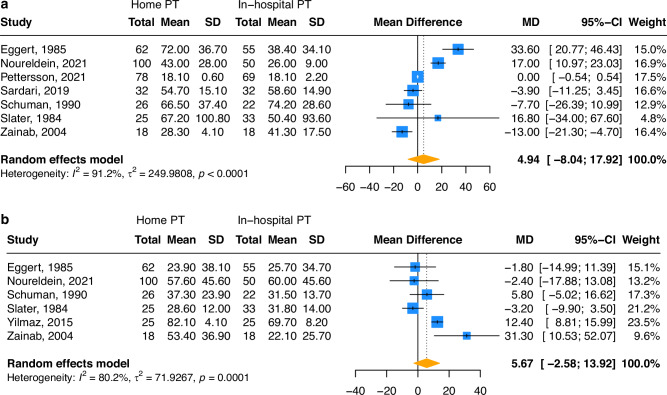
Forest plot meta-analyses effectiveness outcomes. **a** Treatment duration in home phototherapy versus in-hospital phototherapy. **b** Daily serum bilirubin reduction in treatment with home phototherapy versus in-hospital phototherapy. CI confidence interval, SD standard deviation, PT phototherapy.

Data on treatment duration of home phototherapy compared to in-hospital phototherapy were available in seven studies.^24,27,29,32,34,35,37^ There was no statistically significant difference in treatment duration between home phototherapy and in-hospital phototherapy (mean difference (MD) = +4.9 h, 95% CI = –8.0 to +17.9; *I*^*2*^ = 91%; 620 participants) (Fig. 3a).

#### Serum bilirubin reduction in home versus in-hospital phototherapy

Data retrieved from six studies were included in this meta-analysis.^29,32,34–37^ No statistically significant difference was found in daily serum bilirubin reduction between home phototherapy and in-hospital phototherapy (MD = +5.7 μmol/L, 95% CI = −2.6 to +13.9; *I*^*2*^ = 80%; 459 participants) (Fig. 3b).

### Primary outcome - health care costs

Health care costs data were available in 12 studies.^8,26,29–31,35,37,40–43,47^ Due to the heterogeneity of study designs and the diversity in cost measurements, we could not perform a meta-analysis. Five studies included a control group, of which all five showed that home phototherapy was less expensive than in-hospital phototherapy (savings ranging from US$70-US$982 per patient per day).^26,30,31,37,40^ The cost savings in each individual study are detailed in Supplementary Table S7. No studies conducted formal cost-effectiveness analyses.

### Primary outcome - safety

No severe adverse events were reported among 688 neonates treated with home phototherapy from eight studies^24,27,29,35,37,42,46,47^ (Supplementary Table S8). There were no neonates that needed exchange transfusions.^24^ In a few studies, mild adverse events were reported such as vomiting, hyperthermia, restlessness or dehydration.^29,46^ Within individual studies with a control group, no statistically significant differences were found in the occurrence of mild adverse events such as skin rashes, hyperthermia, diarrhea, vomiting, weakness and restlessness between home phototherapy and in-hospital phototherapy.^27,37^ None of the readmitted neonates needed exchange transfusion, or developed ABE or KSD.

### Primary outcome - barriers and facilitators according to parents and health care professionals

Seventeen studies explored perspectives of parents and health care professionals.^8,9,31,32,35,37,40–45,47–51^ Fourteen studies were quantitative studies, two qualitative studies, and one was a mixed-methods study. Three studies primarily focused on health care professionals’ attitudes and experiences,^9,44,50^ six studies evaluated parental experiences,^35,37,45,47,48,51^ and the remaining eight studies evaluated both parental and health care professionals’ experiences.^8,31,32,40–43,49^

A summary of barriers and facilitators according to the TDF domain is provided in Table 2. The most important factors were related to the following domains: knowledge, skills, social/professional role, beliefs about consequences, goals, environmental context and resources, and emotion, and are described below.

**Table 2:** Barriers and facilitators according to parents and healthcare providers in TDF domains.

Domain	Barriers	Facilitators
Knowledge	H: Challenges in accurately informing hospital neonatology and nursing staff^8^ P: Lack of information about duration of treatment^51^	H: Careful screening and selection of appropriate candidates for home PT^38,41,44^ P: Good parent education and availability of (written) information^29,38,41,51^ P: Demonstration on how to use the equipment^9,38^
Skills	P: Difficulties with the equipment, such as struggling to put the eye mask on^9,35,51^	H: An option for nurses who may be looking for additional ways to use their skills^41^ P: Equipment was easy to use^9,34,51^
Social/professional and role and identity	H: Information dissemination and cultural change across a larger tertiary hospital^8^ P: Responsible for your own baby without constant medical supervision^9^	H: Opportunity to offer additional support to a new family^41^ H: Opportunity to develop a bond with the family^49^ H: CHW perception of parental confidence in the provision of home PT^49^ P: Advice of the physician to start with home PT^8^ P: Nurse’s daily visit at home^29,41,43,49^ P: Daily contact with the nurse by phone call^8,9^ P: Support by midwives^31^ P: More comfortable contacting the hospital for advice if parents had concerns^8,51^
Beliefs about capabilities	H: Lack of confidence in CHW skills^49^ P: Parental incapability to comply with instructions^38,42^	H: Trusting parents^38^ H: Ensuring parents will cope with home PT^38^
Beliefs about consequences	H: Concerns about potential consequences of community-based care^8^ H: Concerns about safety^38,42,45^ H: Concerns about the reliability of home blood testing^44^ H: Concerns about lack of compliance^38,45^ H/P: Concerns about equipment failure or damage^38,51^ H: The sun is more effective^42^ H: Lack of monitoring^38^ H: The costs to set-up in the first place^38^ P: Increased work for mothers^29^	H: Staff wellbeing^38^ H: Workload will decrease^38,49^ H: Decreased healthcare expenditure^38^ H: Reduced risks associated with inpatient care^38^ P: Hold and feed infant during PT at home^43^ P: Continuing breastfeeding^37^ H/P: Reduced costs^35,42,44^
Reinforcement		
Intentions		
Goals		H/P: Family centered care^8,9,38^ H/P: Parent empowerment^38^ H/P: Parent autonomy^38^ H/P: Better mother-child bonding^8,9,35,42,51^ H: Improving flow of patients^38^ H: Getting well babies out of the hospital^38,44^
Memory, attention, and decision processes		
Environment context and resources	H: Difficulties with blood drawing at home^29,44^ H: Choosing the location for the therapy is a concern^29^ H: It is not possible to check bilirubin levels as recommended by guidelines^31^ H: Administrative difficulties^44^ H: Insurance reimbursement^44,45^ H/P: Distance from parental home to hospital^35,38^ H: Transportation of the PT device is difficult^49^ P: Having to stay indoors^9^	H: Flexibility in the schedules of healthcare providers^31,41^ H/P: Home environment is good^9,45,47,51^ P: Freedom^9,51^ P: Maintaining routine at home^9,51^
Social influences	H: Lack of use by colleagues^44^	P: Desirable to have to people at home during PT^51^
Optimism		
Emotions	H: Fears that parents would not comply with PT^38,44^ H: Fears that parents would not apply eye shields^44^ H: Fears of overheating or dehydration^44^ H/P: Parental anxiety^44,45,51^ H: Concerns about parental anxiety^44^ P: Parental stress^51^ P: Some parents felt more secure in hospital^51^ P: Insecure about handling and taking care for own newborn^51^	H: Psychological wellbeing of parents: less traumatic, more relaxed, happier^38^ P: Hospitalization is less traumatic^51^ P: Lower stress levels of parents^23,51^ P: Parents felt secure^51^
Behavioral regulation		

*P* parents, *H* healthcare professionals, *CHW* community health workers, *PT* phototherapy.

Knowledge about home phototherapy was seen as an important facilitator by parents, which included demonstration on how to use the phototherapy equipment^32,50^ and the availability of (written) information about phototherapy.^40,41,50,51^ Lack of information, such as missing information about the expected duration of home phototherapy treatment, was mentioned as a barrier.^51^ Effectively informing all health care professionals about the home phototherapy program proved to be challenging. Additionally, health care professionals had reservations about the community’s ability to provide the appropriate care.^8^ Knowledge about careful screening and selection of potentially eligible patients for home phototherapy was reported as a facilitator.^41,44,50^

In the ‘skills’ domain, parents reported barriers to properly administering home phototherapy, including difficulties with the equipment, such as placing the neonate’s eye mask on correctly.^32,35,48,51^ However, ‘the equipment was easy to use’ was reported as a facilitator in several studies, specifically when using the BiliCocoon Bag (NeoMedLight, Villeurbanne, France) or the BiliSoft blanket (GE Healthcare, Chicago, Illinois).^32,34,51^

In the domain ‘social/professional role and identity’, parents viewed the involvement of different health care professionals, such as daily contact with nurses and/or support from midwives, as a key facilitator.^8,31,32,40,41,43,49^ The ability to contact the hospital for advice if parents had concerns was also regarded as a facilitator.^8,51^

Beliefs about the potential adverse consequences of home-based care was seen as an important barrier, including concerns about parental compliance and the safety of the neonate due to insufficient monitoring.^8,42,44,50^ However, positive consequences were also reported by health care professionals, including decreased health care expenditure^42,44,50^ and a decreased workload.^49,50^

In the domain ‘goals’, multiple facilitators were identified. The most commonly mentioned goals of home phototherapy, according to both health care professionals and parents, were family centered care,^8,32,50^ improved parent-child bonding,^8,32,35,42,51^ and discharging otherwise healthy babies from the hospital.^44,50^

In the ‘environmental context and resources’ domain, we found significant barriers, including the travel distance from parental home to the hospital for check-ups,^35,50^ difficulties with blood draws,^31,40,44^ and issues with insurance reimbursement.^44,45^ The most frequently mentioned facilitator in this domain was the benefit of being in one’s own environment.^32,45,47,51^ Furthermore, parents mentioned freedom and the ability to maintain routines at home as facilitators.^32,51^ Health care professionals cited flexibility in their schedules as a facilitator.^31,41^

Important facilitators and barriers were found in the ‘emotional’ domain. (Concern of) parental anxiety was mentioned as a barrier from both parents’ and health care professionals’ perspectives.^44,45,51^ Furthermore, health care professionals mentioned apprehensiveness about parental non-compliance with phototherapy^44,50^ and fears of overheating or dehydration^44^ as barriers. Facilitators included the perception that hospitalization is more traumatic,^51^ and a perceived reduction in parental stress.^23,51^

### Certainty of evidence

The quality of the evidence included in the systematic review was rated using GRADE. For each of the included studies, the evidence-level was determined. The results of these assessment are summarized in Supplementary Table S9. All outcomes were considered to be of low or very low quality.

## Discussion

This comprehensive mixed-methods systematic review and meta-analysis provides a synthesized framework on the best available evidence of home phototherapy use for neonatal hyperbilirubinemia. By pooling data from over four thousand (near) term neonates treated with home phototherapy, we found a mean hospital readmission rate of 3.5%. This did not vary substantially according to the eligibility criteria for home phototherapy used in individual studies. Daily serum bilirubin reduction and treatment duration were similar between home phototherapy and in-hospital phototherapy, and no severe adverse events were reported. All studies that included a control group showed that home phototherapy was less expensive than in-hospital phototherapy. However, formal cost-effectiveness assessments were lacking. These findings suggest that home phototherapy for neonatal hyperbilirubinemia can be an effective and safe alternative to in-hospital phototherapy in a selected low-risk patient group (e.g. gestational age ≥ 35 weeks, postnatal age ≥24 h). Still, the quality of evidence is of low certainty.

To successfully implement home phototherapy, the following considerations should be taken into account, based on insights from 869 health care professionals and 478 parents. First, it is important to thoroughly screen neonates and their parents to determine their suitability for home phototherapy. Second, providing comprehensive education to parents about jaundice, phototherapy, the phototherapy device, and about the process and benefits of home phototherapy is necessary. Finally, maintaining good communication and documentation regarding the treatment and providing a designated contact person for parents or caregivers are essential. Several key barriers must also be addressed. Challenges in home bilirubin quantification need to be mitigated. Concerns from health care professionals about the potential adverse consequences of home-based care can be alleviated through clear (inter-)national guidelines and support structures, such as home visits by (maternity care) nurses or community midwives. Additionally, reducing parental insecurity via offering adequate information, documentation and home support is crucial.

Previous systematic reviews and meta-analyses had contradicting findings and therefore different clinical recommendations.^10–14^ To our knowledge, this study represents the most comprehensive assessment to date regarding home phototherapy for neonatal hyperbilirubinemia. We conducted a highly comprehensive search strategy, covering several electronic databases, reference searches, and citation searches. One of the strengths of this study is that we included all studies designs reporting data on home phototherapy for hyperbilirubinemia in (near) term neonates, including qualitative and mixed-methods studies. Moreover, our study is the first to systematically synthesize the perspectives from both parent(s)/caregiver(s) and health care professionals on home phototherapy based on the Theoretical Domains Framework, providing a broad scope of analysis. We incorporated the GRADE guideline which provided a clear overview of the existing certainty of evidence.

Inherent to a systematic review, our work is limited by the amount and quality of the underlying evidence. Specifically, we were unable to obtain full access to some potentially relevant - but mostly older - studies, despite efforts to mitigate this issue by using available abstracts and contacting authors and journals for access. This may have led to an incomplete representation of the current evidence base. Furthermore, the moderate risk of bias in the included studies and the absence of a control group in most studies needs to be considered when interpreting the results. Between-study heterogeneity in methodology, differences in inclusion criteria for home phototherapy, in phototherapy devices (including variation in irradiance), and in phototherapy treatment thresholds should also be taken into consideration. As most studies included in this review were conducted in high- or upper-middle-income countries, generalizability of the findings to low- or middle-income countries is unclear and likely limited.

## Conclusion

In conclusion, our findings indicate that home phototherapy can be a safe and effective alternative to in-hospital phototherapy for treating hyperbilirubinemia in low-risk (near) term neonates, although the strength of this recommendation is low. Careful eligibility screening and adequate parental education facilitated home phototherapy, while barriers included challenges in healthcare professionals’ concerns, and parental insecurity. Hence, addressing these and other barriers including via enhanced support and accurate home monitoring is recommended. Such aspects should be considered when formulating much-needed guidelines for safe and effective implementation of home phototherapy programs.

## Supplementary information

Supplementary materials

## Data Availability

All data generated or analysed during this study are included in this published article [and its supplementary information files].
