# Supplementary material for: Home phototherapy for neonatal hyperbilirubinemia: a mixed methods systematic review and meta-analysis
Source: Pediatr Res. 2025 Jul 26;99(6):2155–68. doi: 10.1038/s41390-025-04313-5 (PMC13433316; doi:10.1038/s41390-025-04313-5)
Supplement: Supplementary file 1 [file 41390_2025_4313_MOESM1_ESM.docx]

**Supplemental Online Content**

**Supplemental Table S1.** PRISMA checklist

**Supplemental Table S2.** Search terms

**Supplemental Table S3.** Data extraction table

**Supplemental Table S4.** Characteristics of home phototherapy devices

**Supplemental Table S5.** Risk of Bias (Mixed Methods Appraisal Tool)

**Supplemental Table S6.** Results of the effectiveness outcomes

**Supplemental Table S7.** Results of the costs

**Supplemental Table S8.** Results of the safety outcomes

**Supplemental Table S9.** Quality of evidence

**Supplemental Table S1. PRISMA checklist**

| **Section and Topic** | **Item #** | **Checklist item** | **Location where item is reported** |
| --- | --- | --- | --- |
| **TITLE** | | |  |
| Title | 1 | Identify the report as a systematic review. | Title, page 1 |
| **ABSTRACT** | | |  |
| Abstract | 2 | See the PRISMA 2020 for Abstracts checklist. | Abstract, page 3 |
| **INTRODUCTION** | | |  |
| Rationale | 3 | Describe the rationale for the review in the context of existing knowledge. | Introduction: Paragraph 2, page 5 |
| Objectives | 4 | Provide an explicit statement of the objective(s) or question(s) the review addresses. | Introduction: Paragraph 3, page 5 |
| **METHODS** | | |  |
| Eligibility criteria | 5 | Specify the inclusion and exclusion criteria for the review and how studies were grouped for the syntheses. | Methods: type of studies and topic of interest, page 6 |
| Information sources | 6 | Specify all databases, registers, websites, organisations, reference lists and other sources searched or consulted to identify studies. Specify the date when each source was last searched or consulted. | Methods: search strategy and selection criteria, page 6 |
| Search strategy | 7 | Present the full search strategies for all databases, registers and websites, including any filters and limits used. | Methods: search strategy in supplements |
| Selection process | 8 | Specify the methods used to decide whether a study met the inclusion criteria of the review, including how many reviewers screened each record and each report retrieved, whether they worked independently, and if applicable, details of automation tools used in the process. | Methods: search strategy and selection criteria, page 6 |
| Data collection process | 9 | Specify the methods used to collect data from reports, including how many reviewers collected data from each report, whether they worked independently, any processes for obtaining or confirming data from study investigators, and if applicable, details of automation tools used in the process. | Methods: Data extraction, page 7 |
| Data items | 10a | List and define all outcomes for which data were sought. Specify whether all results that were compatible with each outcome domain in each study were sought (e.g. for all measures, time points, analyses), and if not, the methods used to decide which results to collect. | Methods: Data extraction, page 7 |
|  | 10b | List and define all other variables for which data were sought (e.g. participant and intervention characteristics, funding sources). Describe any assumptions made about any missing or unclear information. | Data extraction table in supplements |
| Study risk of bias assessment | 11 | Specify the methods used to assess risk of bias in the included studies, including details of the tool(s) used, how many reviewers assessed each study and whether they worked independently, and if applicable, details of automation tools used in the process. | Methods: quality assessment of included studies, page 7 |
| Effect measures | 12 | Specify for each outcome the effect measure(s) (e.g. risk ratio, mean difference) used in the synthesis or presentation of results. | Methods: data synthesis, page 8 |
| Synthesis methods | 13a | Describe the processes used to decide which studies were eligible for each synthesis (e.g. tabulating the study intervention characteristics and comparing against the planned groups for each synthesis (item #5)). | Methods: data synthesis – paragraph 1, page 8 |
|  | 13b | Describe any methods required to prepare the data for presentation or synthesis, such as handling of missing summary statistics, or data conversions. | Methods: data synthesis – paragraph 1, page 8 |
|  | 13c | Describe any methods used to tabulate or visually display results of individual studies and syntheses. | Methods: data synthesis – paragraph 1, page 8 |
|  | 13d | Describe any methods used to synthesize results and provide a rationale for the choice(s). If meta-analysis was performed, describe the model(s), method(s) to identify the presence and extent of statistical heterogeneity, and software package(s) used. | Methods: data synthesis – paragraph 1, page 8 |
|  | 13e | Describe any methods used to explore possible causes of heterogeneity among study results (e.g. subgroup analysis, meta-regression). | Methods: data synthesis – paragraph 1, page 8 |
|  | 13f | Describe any sensitivity analyses conducted to assess robustness of the synthesized results. | Methods: page 8 |
| Reporting bias assessment | 14 | Describe any methods used to assess risk of bias due to missing results in a synthesis (arising from reporting biases). | Methods: page 8 |
| Certainty assessment | 15 | Describe any methods used to assess certainty (or confidence) in the body of evidence for an outcome. | Methods: page 8 |
| **RESULTS** | | |  |
| Study selection | 16a | Describe the results of the search and selection process, from the number of records identified in the search to the number of studies included in the review, ideally using a flow diagram. | Results: paragraph 1, page 9 |
|  | 16b | Cite studies that might appear to meet the inclusion criteria, but which were excluded, and explain why they were excluded. | Results: paragraph 1 and Figure 1, page 9 |
| Study characteristics | 17 | Cite each included study and present its characteristics. | Results: study characteristics and Table 1, page 9 |
| Risk of bias in studies | 18 | Present assessments of risk of bias for each included study. | Results: Quality of the studies and S3 Appendix, page 9-10 |
| Results of individual studies | 19 | For all outcomes, present, for each study: (a) summary statistics for each group (where appropriate) and (b) an effect estimate and its precision (e.g. confidence/credible interval), ideally using structured tables or plots. | Results: page 10 |
| Results of syntheses | 20a | For each synthesis, briefly summarise the characteristics and risk of bias among contributing studies. | Results: page 9 |
|  | 20b | Present results of all statistical syntheses conducted. If meta-analysis was done, present for each the summary estimate and its precision (e.g. confidence/credible interval) and measures of statistical heterogeneity. If comparing groups, describe the direction of the effect. | Results: page 10-12 |
|  | 20c | Present results of all investigations of possible causes of heterogeneity among study results. | Results: page 10-12 |
|  | 20d | Present results of all sensitivity analyses conducted to assess the robustness of the synthesized results. | Not applicable |
| Reporting biases | 21 | Present assessments of risk of bias due to missing results (arising from reporting biases) for each synthesis assessed. | Not applicable |
| Certainty of evidence | 22 | Present assessments of certainty (or confidence) in the body of evidence for each outcome assessed. | Results: page 10-12 |
| **DISCUSSION** | | |  |
| Discussion | 23a | Provide a general interpretation of the results in the context of other evidence. | Discussion: page 15 |
|  | 23b | Discuss any limitations of the evidence included in the review. | Discussion: page 16 |
|  | 23c | Discuss any limitations of the review processes used. | Discussion: page 16 |
|  | 23d | Discuss implications of the results for practice, policy, and future research. | Discussion: page 15 |
| **OTHER INFORMATION** | | |  |
| Registration and protocol | 24a | Provide registration information for the review, including register name and registration number, or state that the review was not registered. | Methods: page 6 |
|  | 24b | Indicate where the review protocol can be accessed, or state that a protocol was not prepared. | Not applicable |
|  | 24c | Describe and explain any amendments to information provided at registration or in the protocol. | Not applicable |
| Support | 25 | Describe sources of financial or non-financial support for the review, and the role of the funders or sponsors in the review. | Page 18 |
| Competing interests | 26 | Declare any competing interests of review authors. | Page 18 |
| Availability of data, code and other materials | 27 | Report which of the following are publicly available and where they can be found: template data collection forms; data extracted from included studies; data used for all analyses; analytic code; any other materials used in the review. | Supplements |

*From:*  Page MJ, McKenzie JE, Bossuyt PM, Boutron I, Hoffmann TC, Mulrow CD, et al. The PRISMA 2020 statement: an updated guideline for reporting systematic reviews. BMJ 2021;372:n71. doi: 10.1136/bmj.n71

For more information, visit: <http://www.prisma-statement.org/>

**Supplemental Table S2. Search terms**

| **Database searched** | **Platform** | **Years of coverage** | **Records** | **Records after duplicates removed** |
| --- | --- | --- | --- | --- |
| Embase | Embase.com | 1971 - Present | 324 | 185 |
| Medline ALL | Ovid | 1946 - Present | 219 | 219 |
| Web of Science Core Collection* | Web of Knowledge | 1975 - Present | 115 | 19 |
| Cochrane Central Register of Controlled Trials | Wiley | 1992 - Present | 22 | 2 |
| CINAHL | EBSCO | 1982 - Present | 77 | 18 |
| Additional Search Engines: Google Scholar | | | 100 | 66 |
| **Total** | | | **857** | **509** |

*Science Citation Index Expanded (1975-present) ; Social Sciences Citation Index (1975-present) ; Arts & Humanities Citation Index (1975-present) ; Conference Proceedings Citation Index- Science (1990-present) ; Conference Proceedings Citation Index- Social Science & Humanities (1990-present) ; Emerging Sources Citation Index (2015-present)

**Embase**

('phototherapy'/exp OR 'therapy'/de OR (phototherap* OR lighttherap* OR ((photo OR light) NEXT/1 (therap*))):ab,ti,kw) **AND** ('hyperbilirubinemia'/de OR 'jaundice'/exp OR 'neonatal hyperbilirubinemia'/de OR (hyperbilirubinem* OR hyperbilirubinaem* OR jaundice*):ab,ti,kw) **AND** ('child'/exp OR 'neonatal hyperbilirubinemia'/de OR 'infant'/exp OR (newborn* OR new*-born* OR neonat* OR baby OR babies OR infant* OR child*):ab,ti,kw) **AND** ('outpatient'/de OR 'home care'/exp OR 'home'/exp OR (outpatient* OR out*-patient* OR home OR homes* OR homecare* OR homesetting* OR ((domicil* OR domestic* OR self*) NEAR/3 (care*))):ab,ti,kw)

**Medline**

(exp Phototherapy/ OR therapy.fx. OR (phototherap* OR lighttherap* OR ((photo OR light) ADJ (therap*))).ab,ti,kf.) AND (Hyperbilirubinemia/ OR exp Jaundice/ OR exp Hyperbilirubinemia, Neonatal/ OR (hyperbilirubinem* OR hyperbilirubinaem* OR jaundice*).ab,ti,kf.) AND (exp Child/ OR exp Hyperbilirubinemia, Neonatal/ OR exp Infant/ OR (newborn* OR new*-born* OR neonat* OR baby OR babies OR infant* OR child*).ab,ti,kf.) AND (exp Outpatients/ OR exp Home Care Services/ OR exp Home Environment/ OR (outpatient* OR out*-patient* OR home OR homes* OR homecare* OR homesetting* OR ((domicil* OR domestic* OR self*) ADJ3 (care*))).ab,ti,kf.)

**Cochrane**

((phototherap* OR lighttherap* OR ((photo OR light) NEXT/1 (therap*))):ab,ti,kw) **AND** ((hyperbilirubinem* OR hyperbilirubinaem* OR jaundice*):ab,ti,kw) **AND** ((newborn* OR (new* NEXT/1 born*) OR neonat* OR baby OR babies OR infant* OR child*):ab,ti,kw) **AND** ((outpatient* OR (out* NEXT/1 patient*) OR home OR homes* OR homecare* OR homesetting* OR ((domicil* OR domestic* OR self*) NEAR/3 (care*))):ab,ti,kw)

**Web of Science**

TS=(((phototherap* OR lighttherap* OR ((photo OR light) NEAR/1 (therap*)))) AND ((hyperbilirubinem* OR hyperbilirubinaem* OR jaundice*)) AND ((newborn* OR new*-born* OR neonat* OR baby OR babies OR infant* OR child*)) AND ((outpatient* OR out*-patient* OR home OR homes* OR homecare* OR homesetting* OR ((domicil* OR domestic* OR self*) NEAR/2 (care*)))))

**Cinahl**

(MH Phototherapy+ OR TI(phototherap* OR lighttherap* OR ((photo OR light) N1 (therap*))) OR AB(phototherap* OR lighttherap* OR ((photo OR light) N1 (therap*)))) AND (MH Hyperbilirubinemia+ OR MH Jaundice+ OR MH Hyperbilirubinemia, Neonatal+ OR TI(hyperbilirubinem* OR hyperbilirubinaem* OR jaundice*) OR AB(hyperbilirubinem* OR hyperbilirubinaem* OR jaundice*)) AND (MH Child+ OR MH Hyperbilirubinemia, Neonatal+ OR MH Infant+ OR TI(newborn* OR new*-born* OR neonat* OR baby OR babies OR infant* OR child*) OR AB(newborn* OR new*-born* OR neonat* OR baby OR babies OR infant* OR child*)) AND (MH Outpatients+ OR MH Outpatient Service+ OR MH Home Health Care+ MH Home Environment+ OR TI(outpatient* OR out*-patient* OR home OR homes* OR homecare* OR homesetting* OR ((domicil* OR domestic* OR self*) N2 (care*))) OR AB(outpatient* OR out*-patient* OR home OR homes* OR homecare* OR homesetting* OR ((domicil* OR domestic* OR self*) N2 (care*))))

**Google Scholar**

phototherapy|lighttherapy|”photo|light therapy” hyperbilirubinemia|hyperbilirubinaemia|jaundice newborn|infant|child outpatient|”out patient”|home|homecare|homesetting

**Supplemental Table S3. Data extraction table**

| Data extraction file |  |
| --- | --- |
| Who completed the data extraction |  |
| Date of extraction |  |
| Data item numbers to discuss |  |
| Title of article |  |
| General data items paper |  |
| (1) First author’s name |  |
| (2) His or her affiliation |  |
| (3) Country where study was performed |  |
| (4) Publication year |  |
| (5) Access information (URL of doi) |  |
| (6) Study design |  |
| Data home phototherapy as intervention |  |
| (7) Number of infants who received PT at home |  |
| (8) Number of infants who received PT in-hospital |  |
| (9) Inclusion criteria |  |
| (10) Exclusion criteria |  |
| (11) Phototherapy device (name, manufacturer, type of light, type of material) |  |
| (12) Professionals involved e.g. pediatrician, midwives, maternity care assistants and nurses |  |
| (13) Information and instructions regarding PT at home |  |
| (14) Effectiveness: rate of bilirubin reduction |  |
| (15) Effectiveness: PT duration |  |
| (16) Effectiveness: hospital (re)admission rate |  |
| (17) Safety: complications of PT treatment |  |
| (18) Safety: exchange transfusion |  |
| (19) Safety: acute encephalopathy |  |
| (20) Safety: kernicterus spectrum disorder |  |
| (21): Safety: school performance, mortality |  |
| (22) Characteristics neonates (ethnicity, gestational age, cause for severe hyperbilirubinemia, bilirubin value at start of treatment) |  |
| (23) Characteristics parents (single parenthood, social economic status, highest education degree achieved, first child yes/no, ethnicity) |  |
| Barriers and facilitators |  |
| (24) Facilitator(s) as indicated by original study |  |
| (25) Barrier(s) as indicated by original study |  |
| Parental and health care professional experience |  |
| (26) Number of parents/other caregivers |  |
| (27) Parental experience;  Parental distress, feeling comfortable with PT, information and instructions regarding PT at home, user-friendliness of PT device, availability of help in case of questions or problems |  |
| (27) Number of healthcare professionals |  |
| (28) Health care professional experience;  Contact with the parents, satisfaction |  |

**Supplemental Table S4. Characteristics of home phototherapy devices**

| Author and year | home phototherapy device* | Light irradaiance | Professionals involved | Information and instructions |
| --- | --- | --- | --- | --- |
| ADLINA ET AL, 2007 [38] | The bluelite mobile home phototherapy unit (locally designed, SIRIM approved and internationally patented product) | Not reported | Pediatrician | Written instructions on the usage of the equipment |
| chang et al, 2020 [39] | BiliBed (Medela, McHenry, Illinois, USA) | 36 uW/cm/nm | Pediatrician, pediatric nurse, company staff | Not reported |
| coquery et al, 2022 [28] | Bilicocoon Bag (NeoMedLight) | 35 uW/cm/nm | Pediatrician, pediatric nurse | Verbal instructions by pediatric nurse |
| dortch et al, 1986 [40] | Cavitron and Minolta | 6 – 8 uW/cm/nm | Physician and pediatric nurse | Verbal instructions by pediatric nurse and written instructions on the phototherapy treatment and jaundice |
| eggert et al, 1985  [29] | Artificial light | Radiant flux > 500 uW/cm/nm | Physician and pediatric nurse | Verbal instructions by pediatric nurse and written instruction on phototherapy treatment |
| ellis et al, 1985 [41] | Portable lights from Rocky Mountain Medical Corp and from Olympic Medical | Not reported | Hospital based: pediatricians, neonatologists, nurses  Private pediatrics practice: pediatrician  Independent nurse practice: nurses with back up from local pediatric and neonatologist | Verbal instructions by pediatric nurse and written instructions |
| grabert et al, 1985  [42] | Olympic Bili-Lite (Seattle, WA, USA) with one half Cool White Light and one half special Blue Bulbs | Not reported | Pediatrician, pediatric nurse | Verbal instructions by pediatric nurse and written instructions |
| hamelin et al, 1998  [43] | Ohmeda Biliblanket  Light irradiance of 33 to 35 uW/cm/nm | 33 to 35 uW/cm/nm | Public health nurse, pediatrician | Verbal instructions (including demonstration) and written instructions |
| Jackson et al, 2000  [8] | BiliBlanket | 33 to 35 uW/cm/nm | Pediatrician, nurses | Verbal instructions by pediatric nurse |
| khAjehei et al, 2022  [31] | BiliSoft blanket (GE Healthcare, Chicago, Illinois, USA) – a fibre-optic light-emitting diode blanket | 35 uW/cm/nm | Pediatricians, midwives | Verbal instructions, written information and a video link |
| Meropol et al, 1993  [44] | Not reported | Not reported | Pediatric nurse, researcher | Verbal information and instructions by pediatric nurse  Nurse care session of about 45 – 60 min was held at home |
| noureldein et al, 2021 [32] | BiliCocoon Bag (NeoMedLight) | 35 uW/cm/nm | Community outreach nursing team, consultant pediatrician | Verbal instructions |
| Orringer et al, 2022 [33] | Biliblanket | 33 to 35 uW/cm/nm | Not reported | Not reported |
| Pettersson et al, 2021 [24] | BiliSoft blanket GE Healthcare, Chicago, Illinois, USA) – a fibre-optic light-emitting diode blanket  Light irradiance of 35 uW/cm/nm | 35 uW/cm/nm | Physician | Written information |
| Rogerson et al, 1985 [45] | Initially a pair of fluorescent lights, each consisting of two 20-watt, 20-inch tubes were used.  8 years ago switch to standard bilirubin fixture (Olympic Bililite) with eight 40-watt tubes. | Not reported | Physician, pediatric nurse, laboratory staff | Verbal instructions by pediatric nurse |
| Sardari et al, 2019 [27] | Not reported | Not reported | Not reported | Checklist |
| Schuman et al, 1990 [34] | Fiberoptic phototherapy  Conventional phototherapy (bililight = E and B electronics – two white fluorescent and two special blue PT bulbs | 8uW/cm2/nm to 10 uW/cm2/nm  10uW/cm2/nm to 12 uW/cm2/nm | Homecare agency, physician/pediatrician, laboratory staff | Verbal instructions by homecare agency |
| Slater et al, 1984 [35] | Olympic Bililite | Not reported | 4 specially trained nurses, study staff, primary care assistant | Verbal instructions by pediatric nurse and written instructions |
| thakkar et al, 2019 [47] | Biliblanket  BiliSoft | 33 to 35 uW/cm/nm  35 uW/cm/nm | Outreach team | Not reported |
| zainab et al, 2004 [37] | Mobile home unit (Bluelite Portable Light) | 15.5 uW/cm/nm | Pediatrician | Demonstration on the use of the equipment |
| zanardo et al, 2021 [48] | The neonatal phototherapy lamp uses latest LED technology to produce blue therapeutic light (peak 460  nm) transmitted through a flexible fiber optic cable to a lightening element of small dimensions (area pad  efficacy 110x160 mm) placed in contact with the skin of the neonate | 45 µW/cm2 /nm | Neonatal staff | Verbal instructions / demonstration (how to use the equipment) |

**Supplemental Table S5. Risk of Bias (Mixed Methods Appraisal Tool)**

**S5.1.** Critical appraisal of qualitative studies

| Study details | screening questions | | Qualitative studies questions | | | | | | mmat score |
| --- | --- | --- | --- | --- | --- | --- | --- | --- | --- |
| AUTHOR | S1. Are there clear research questions? | S2. Do the collected data allow to address the research questions? | 1.1. Is the qualitative approach appropriate to answer the research question? | 1.2. Are the qualitative data collection methods adequate to address the research question? | 1.3. Are the findings adequately derived from the data? | 1.4. Is the interpretation of results sufficiently substantiated by data? | 1.5. Is there coherence between qualitative data sources, collection, analysis and interpretation? | |  |
| ANDERSON ET AL, 2023 [50] | Yes | Yes | Yes | Yes | N/A | No | | Yes | 60% (Moderate) |
| Pettersson 2022 [26] | Yes | Yes | Yes | Yes | Yes | Yes | | Yes | 100% (High) |
|  |  |  |  |  |  |  | |  |  |

**S5.2.** Critical appraisal of quantitative randomized controlled trials

| Study details | screening questions | | quantitative randomized controlled trials questions | | | | | | mmat score |
| --- | --- | --- | --- | --- | --- | --- | --- | --- | --- |
| AUTHOR | S1. Are there clear research questions? | S2. Do the collected data allow to address the research questions? | 2.1. Is the randomization appropriately performed? | 2.2. Are the groups comparable outcome data? | 2.3. Are there complete outcome data? | 2.4. Are outcome assessors blinded to the intervention provided? | 2.5. Did the participants adhere to the assigned intervention? | |  |
| namnabati 2019 [23] | Yes | Yes | No | Yes | Yes | No | | Yes | 60% (Moderate) |
| PETTERSSON 2021 [24] | Yes | Yes | N/A | Yes | Yes | N/A | | Yes | 60% (Moderate) |
| PETTERSSON  2021 (2) [25] | Yes | Yes | N/A | Yes | Yes | N/A | | Yes | 60% (Moderate) |
| PETTERSSON 2023 [26] | Yes | Yes | N/A | Yes | Yes | N/A | | Yes | 60% (Moderate) |
| SARDARI 2019 [27] | Yes | Yes | No | No | Yes | No | | Yes | 40% (Low) |

**S5.3.** Critical appraisal of quantitative non-randomized studies

| Study details | screening questions | | quantitative non-randomized studies questions | | | | | | mmat score |
| --- | --- | --- | --- | --- | --- | --- | --- | --- | --- |
| AUTHOR | S1. Are there clear research questions? | S2. Do the collected data allow to address the research questions? | 3.1. Are the participants representative of the target population? | 3.2. Are the measurements appropriate regarding both the outcome and the intervention? | 3.3. Are there complete outcome data? | 3.4. Are the confounders accounted for in the design and analysis? | 4.5. During the study period, is the intervention administered as intended? | |  |
| CNOSSEN 2024 [9] | Yes | Yes | Yes | Yes | Yes | Yes | | Yes | 100% (High) |
| coquery 2022 [28] | Yes | Yes | Yes | No | Yes | Yes | | Yes | 80% (Moderate) |
| Khajehei  2022 [31] | Yes | Yes | Yes | No | Yes | Yes | | Yes | 80% (Moderate) |
| noureldein 2021 [32] | Yes | Yes | Yes | Yes | Yes | Yes | | Yes | 100% (High) |
| Orringer 2022 [33] | Yes | Yes | Yes | No | Yes | Yes | | Yes | 80% (Moderate) |
| schuman 1990 [34] | Yes | Yes | Yes | Yes | Yes | Yes | | Yes | 100% (High) |
| SLATER  1984 [35] | Yes | Yes | Yes | Yes | Yes | Yes | | Yes | 100% (High) |
| YILMAZ  2015 [36] | Yes | Yes | Yes | Yes | Yes | No | | Yes | 80% (Moderate) |
| Zainab 2004 [37] | Yes | Yes | Yes | Yes | Yes | Yes | | Yes | 100% (High) |

**S5.4.** Critical appraisal of quantitative descriptive studies

| Study details | screening questions | | quantitative descriptivestudies questions | | | | | | mmat score |
| --- | --- | --- | --- | --- | --- | --- | --- | --- | --- |
| AUTHOR | S1. Are there clear research questions? | S2. Do the collected data allow to address the research questions? | 4.1. Is the sampling strategy relevant to address the research question? | 4.2. Is the sample representative of the target population? | 4.3. Are the measurements appropriate? | 4.4. Is the risk of nonresponse bias low? | 4.5. Is the statistical analysis appropriate to answer the research question? | |  |
| Adlina  2007 [38] | Yes | Yes | Yes | Yes | No | No | | No | 40% (Low) |
| chang  2020 [39] | Yes | Yes | Yes | Yes | Yes | Yes | | Yes | 100%(High) |
| dortch  1986 [40] | Yes | Yes | Yes | Yes | No | No | | No | 40% (Low) |
| eggert  1985 [29] | Yes | Yes | Yes | Yes | Yes | Yes | | Yes | 100% (High) |
| ellis  1985 [41] | Yes | Yes | N/A | N/A | N/A | N/A | | N/A | 0% (Very Low) |
| grabert 1985 [42] | Yes | Yes | Yes | Yes | Yes | Yes | | Yes | 100% (High) |
| hamelin 1988 [43] | Yes | Yes | Yes | Yes | No | Yes | | No | 60% (Moderate) |
| jackson  2000 [8] | Yes | Yes | Yes | Yes | Yes | Yes | | Yes | 100% (High) |
| meropol  1993 [44] | Yes | Yes | Yes | Yes | Yes | Yes | | Yes | 100% (High) |
| rogerson 1985 [45] | Yes | Yes | No | No | Yes | Yes | | Yes | 60% (Moderate) |
| saeid 2023 [46] | Yes | Yes | Yes | Yes | Yes | Yes | | No | 80% (Moderate) |
| thakkar 2019 [47] | Yes | Yes | Yes | Yes | No | Yes | | No | 60% (Moderate) |
| ZANARDO  2021 [48] | Yes | Yes | Yes | Yes | No | Yes | | Yes | 80% (Moderate) |

**S5.5.** Critical appraisal of mixed methods studies

| Study details | screening questions | | mixed methods studies questions | | | | | mmat score |
| --- | --- | --- | --- | --- | --- | --- | --- | --- |
| AUTHOR | S1. Are there clear research questions? | S2. Do the collected data allow to address the research questions? | 1.1. Is there an adequate rationale for using a mixed methods design to address the research question? | 1.2. Are the different components of the study effectively integrated to answer the research question? | 1.3. Are the outputs of the integration of qualitative and quantitative components adequately interpreted? | 1.4. Are divergences and inconsistencies between quantitative and qualitative results adequately addressed? | 1.5. Do the different components of the study adhere to the quality criteria of each tradition of the methods involved? |  |
| jahan 2023 [49] | Yes | Yes | No | No | No | No | Yes | 20% (Very Low) |

**Supplemental Table S6. Results of the effectiveness outcomes**

**Table 6A.** Data on effectiveness of home phototherapy from (non) randomized studies.

| Source | sample size hpt | sample size ipt | daily bilirubin reduction hpt  (Mean, sd in μmol/L) | daily bilirubin reduction ipt  (MEAN, SD in μmol/L) | duration hpt  (MEAN, SD in hours) | duration ipt (MEAN, SD in hours) | readmission rate HPT |
| --- | --- | --- | --- | --- | --- | --- | --- |
| eggert et al, 1985 [29] | 62 | 55 | 23.9, 38.1 | 25.7, 34.7 | 72.0, 36.7 | 38.4, 34.1 | 0 (0%) |
| noureldein et al, 2021 [32] | 100 | 50 | 57.6, 45.6 | 60.0, 45.6 | 43.0, 28.0 | 26.0, 9.0 | 3 (3%) |
| Pettersson et al, 2021 [24] | 78 | 69 | NR | NR | MEDIAN 18.1 (range 15 – 18) | 18.5 hours (range 16 – 28) | 3 (3.8%) |
| sardari et al, 2019 [27] | 32 | 32 | NR | NR | 54.7, 15.1 | 58.6, 14.9 | 2 (6.2%) |
| schuman et al, 1990 [34] | 22 | 26 | 37.3, 23.9 | 31.5, 13.7 | 66.5, 37.4 | 74.2, 28.6 | NR |
| slater et al, 1984 [35] | 25 | 33 | 28.6, 12.0 | 31.8, 14.0 | 67.2, 100.8 | 50.4, 93.6 | 2 (8%) |
| yilmaz et al, 2004 [36] | 25 | 25 | 82.1, 4.1 | 69.7, 8.2 | NR | NR | NR |
| zainab et al, 2004 [37] | 18 | 18 | 53.4, 36.9 | 22.1, 25.7 | 28.3, 8.9 | 41.3, 17.5 | 0 (0%) |

Abbreviations. NR, not reported; HPT, home phototherapy; IPT, in-patient phototherapy; SD, standard deviation.

**Supplemental Table S6. Results of the effectiveness outcomes**

| Source | sample size hpt | daily bilirubin reduction hpt  (Mean, sd in mmol/l) | duration hpt  (MEAN, SD in hours) | readmission rate |
| --- | --- | --- | --- | --- |
| adlina et al, 2007 [38] | 1297 | 29.5, 13.8 | 84.0,  NR | 15 (1.6%) |
| Chang et al, 2020 [39] | 1324 | 33.6, 24.0 | 53.0,  NR | 25 (1.9%) |
| COQuery et al, 2022 [28] | 39 | NR | 33/39: 1x12 hours 5/39: 2x12 hours 1/39: 3x12 hours | 2 (5.1%) |
| dortch et al, 1986 [40] | 41 | NR | NR | 2 (4.9%) |
| grabert et al, 1985 [42] | 260 | 22.6, NR | 58.6, NR | 17 (6.5%) |
| hamelin et al, 1998 [43] | 26 | NR | 63 hours,  NR | 1 (3.8%) |
| Jackson et al, 2000 [8] | 32 | 30.4, NR | 62, NR | 1 (3.1%) |
| khajehei et al, 2022 [31] | 400 | NR | NR | 1 (0.25%) |
| Orringer et al, 2022 [33] | 159 | NR | NR | 6 (4%) |
| rogerson et al, 1985 [45] | 86 | NR | NR | 5 (5.8%) |
| saeid et al, 2023 [46] | 203 | Before PT: 244.5, 41.2 After PT: 138.7, 39.2 | NR | NR |
| thakkar et al, 2019 [47] | 10 | NR | 55.2,  NR | NR |
| zanardo et al, 2021 [48] | 30 | NR | NR | 3 (10%) |
|  |  |  |  |  |

Abbreviations. NR, not reported; HPT, home phototherapy; IPT, in-patient phototherapy; SD, standard deviation.

**Supplemental Table S7. Results of the costs**

| Source | COSTS HPT | COSTS IPT | SAVINGS |
| --- | --- | --- | --- |
| DORTCH ET AL, 1986 [40] | Range: US$75 to US$100 per day | Range: US$300 to US$750 per day |  |
| eggert et al, 1985 [29] |  |  | Savings: US$290 per patient treated  Estimated yearly savings: US$73.152 |
| ELLIS ET AL, 1985 [41] | Range: US$50 to US$250 per day |  |  |
| GRABERT ET AL, 1985 [42] |  |  | Saving: US$430 per patient treated |
| hamelin et al, 1998 [43] |  |  | Estimated yearly savings: US$40.000 |
| JACKSON ET AL, 2000 [8] | Hospital PT: AU$68.690  Recurring costs: AU$41.862  Non-recurring: AU$10.203 |  |  |
| KAVOSI et al, 2023 [30] | US$196,52 per patient | US$397,85 per patient | Savings: US$200,33 per patient treated |
| KHAJEHEI et al, 2022 [31] | AU$640 per day | AU$2100 per day | Savings: AU$1460 per day per patient |
| Pettersson et al, 2021 [42] | €337 per patient | €1156 per patient | Savings: €819 per patient treated (95% CI €613–1025) |
| slater et al, 1984 [35] |  |  | Savings: US$225 per day per patient (range 450 to 900) |
| THAKKAR ET AL, 2019 [47] |  |  | Savings: £185 GBP per day per patient |
| zainab et al, 2004 [37] | RM 60 per day | RM 400 per day | Savings: RM 340 per day |

Abbreviations. NR, Not reported; HPT, home phototherapy; IPT, in-patient phototherapy; CI, Confidence Interval; US, United States, AU, Australian; GBP, Great British Pound; RM, Malaysian Ringgit.

**Supplemental Table S8. Results of the safety outcomes**

| Source | sample size hpt | sample size ipt |  | MILD COMPLICATIONS HPT | MILD COMPLICATIONS IPT | SEVERE COMPLICATIONS HPT | SEVERE COMPLICATIONS IPT |
| --- | --- | --- | --- | --- | --- | --- | --- |
| eggert et al, 1985 [29] | 62 | 55 |  | Vomiting (5), Infrequent stools (1) | NR | NR | NR |
| GRABERT ET AL, 1985 [42] | 260 | NR |  | 0 (0%) | NR | NR | NR |
| Pettersson et al, 2021 [24] | 78 | 69 |  | NR | NR | 0 (No intravenous immunoglobulin or blood exchange transfusion) | 0 (No intravenous immunoglobulin or blood exchange transfusion) |
| SAEID ET AL, 2023 [46] | 203 | NR |  | Hyperthermia (2), Restlessness (3), Improper technique (1), dehydration (1) | NR | NR | NR |
| SARDARI ET AL, 2019 [27] | 32 | 32 |  | Skin rashes (13),  Diarrhea (3), Fever (1), Weakness (6),  Weak lactation (7),  Restlessness (12) | Skin rashes (16),  Diarrhea (3), Fever (3), Weakness (10),  Weak lactation (13), Restlessness (8) | NR | NR |
| slater et al, 1984 [35] | 25 | 33 |  | Mild heel cellulitis (1) | 0 | NR | NR |
| THAKKAR ET AL, 2019 [47] | 10 | NR |  | 0 | NR | NR | NR |
| zainab et al, 2004 [37] | 18 | 18 |  | Skin rash (1), Diarrhea (3) | Skin rash (2), Diarrhea (12) | NR | NR |

Abbreviations. NR, not reported; HPT, home phototherapy; IPT, in-patient phototherapy

**Supplemental Table S9. Quality of evidence**

| **Certainty assessment** |  |  |  |  |  |  | **No. of patients** |  |  | **Certainty** |
| --- | --- | --- | --- | --- | --- | --- | --- | --- | --- | --- |
|  | **Study design** | **Risk of bias** | **Inconsistency** | **Indirectness** | **Imprecision** | **Publication bias** | **Home phototherapy** | **In-hospital phototherapy** | **Mean difference (95% CI)** |  |
| **Readmission rate**  N = 17 | Randomized studies, non-randomized studies and quantitative descriptive studies | Serious | Not serious | Not serious | Not serious | Detected | 4010 neonates |  | 3.5% (2.2 – 5.3) | ⨁⨁◯◯    Low |
| **Bilirubin reduction**  N = 6 | Non-randomized studies | Serious | Serious | Not serious | Serious | Detected | 341 neonates | 279 neonates | +5.7 μmol/L,(-2.6 to +13.9) | ⨁◯◯◯  Very low |
| **Phototherapy duration**  N = 7 | Randomized studies and non-randomized studies | Not serious | Serious | Not serious | Serious | Detected | 256 neonates | 203 neonates | +4.9 hours (-8.0 to +17.9) | ⨁◯◯◯  Very low |
| **Adverse outcomes**  N = 8 | Randomized studies, non-randomized studies and quantitative descriptive studies | Serious | Serious | Serious | Serious | Undetected |  |  |  | ⨁◯◯◯  Very low |
| **Costs**  N = 12 | Randomized studies, non-randomized studies and quantitative descriptive studies | Serious | Serious | Serious | Serious | Undetected |  |  |  | ⨁◯◯◯  Very low |
